# Association between physical performance and cardiovascular events in patients with coronary artery disease: protocol for a meta-analysis

**DOI:** 10.1186/s13643-016-0206-8

**Published:** 2016-02-18

**Authors:** Shuhei Yamamoto, Takayoshi Yamaga, Yasunari Sakai, Takaaki Ishida, Saki Nakasone, Masayoshi Ohira, Erika Ota, Rintaro Mori

**Affiliations:** Department of Rehabilitation, Shinshu University Hospital, 3-1-1 Asahi, Matsumoto-shi, Nagano, 390-8621 Japan; Department of Health Policy, National Center for Child Health and Development, Tokyo, Japan; Department of Physical Therapy, School of Health Sciences, Shinshu University, Nagano, Japan

**Keywords:** Coronary artery disease, Physical performance, Physical functions, Meta-analysis, Protocol, Secondary prevention

## Abstract

**Background:**

Physical performance such as muscle strength or walking speed of patients with coronary artery disease (CAD) is lower than that of people who do not have CAD and is related to mortality and re-admission rates. Recent studies have shown that skeletal muscle strength, such as grip strength, was closely associated with cardiac events. Physical performance testing is quick, safe, and inexpensive and provides a reliable assessment tool for routine clinical practice. The aim of this meta-analysis is to clarify the association between physical performance testing and the risk of cardiovascular events and mortality.

**Methods/design:**

This meta-analysis will include male and female participants of any age in community settings who have a history of the following conditions or procedures: myocardial infarction, or coronary revascularization (coronary artery bypass grafting, percutaneous transluminal coronary angioplasty, or coronary artery stent), angina pectoris, heart failure, heart transplant, or coronary artery disease defined by angiography. We will search EMBASE and MEDLINE, PubMed, and the Cochrane Library with no limitations on date, language, document type, or publication status. Identified studies will be prospective and retrospective cohort studies. Physical performance will be defined as upper extremity strength, lower extremity strength, walking speed, or other performance scale. Six review authors will independently extract study characteristics from included studies. Participants will be divided into subgroups according to age (middle-aged <65 years and elderly ≥65 years), diagnosis (coronary artery disease and heart failure) and follow-up time (up to 12 months and over 12 months). We will pool hazard ratios of Cox proportional hazard models after logarithmic transformation and perform the meta-analysis by using inverse-variance method.

**Discussion:**

To our knowledge, this meta-analysis will be the first report to assess the association between physical performance and cardiovascular events in CAD patients. We hope that these findings may help to estimate the prognosis for CAD patients in clinical practice.

**Systematic review registration:**

PROSPERO CRD42015020886.

## Background

As the life expectancy of patients with heart disease has improved in recent years [1], secondary prevention of re-admission and mortality of these patients is becoming an important issue. Previous studies have shown that circulating biomarkers (e.g., brain natriuretic peptide) [2] and cardiac functions obtained by echocardiography testing (e.g., left ventricular ejection fraction) [3, 4] affect the mortality of these patients. Additionally, recent studies have shown that decreased physical performance such as muscle strength or walking speed was closely related to mortality and cardiovascular events [5, 6]. That physical performance of patients with coronary artery disease (CAD) decreases to approximately 70 % relative to that of people who do not have CAD [7] and is related to mortality and re-admission rates [8–10]. Our previous study showed that a decrease in the walking speed of CAD patients was a strong prognostic indicator of cardiovascular events [5]. Furthermore, deterioration in skeletal muscle strength such as grip strength [11] or leg strength [6, 12] was closely associated with cardiac events in CAD patients. Physical performance testing offers a fast, safe, affordable, and reliable tool for clinical practice. Therefore, further development of physical performance testing is needed. However, the relationship between physical performance testing and cardiovascular events differs by diagnosis and age. For this reason, it is necessary to divide patients into age groups (middle-aged and elderly) or diagnosis groups (CAD and heart failure) in order to verify the association between physical performance and cardiovascular events.

The aim of this meta-analysis is to clarify the association between physical performance testing and risk of cardiovascular events and mortality.

## Methods/design

This meta-analysis was registered with PROSPERO (our registration number: CRD42015020886). We based the review methods on the guidelines of Meta-analysis of Observational Studies in Epidemiology (MOOSE) [13], Preferred Reporting Items for Systematic Reviews and Meta-analyses (PRISMA) [14], and Preferred Reporting Items for Systematic Reviews and Meta-Analyses Protocol (PRISMA-P) statement [15].

### Search methods for identification of studies

**Fig. 1:**
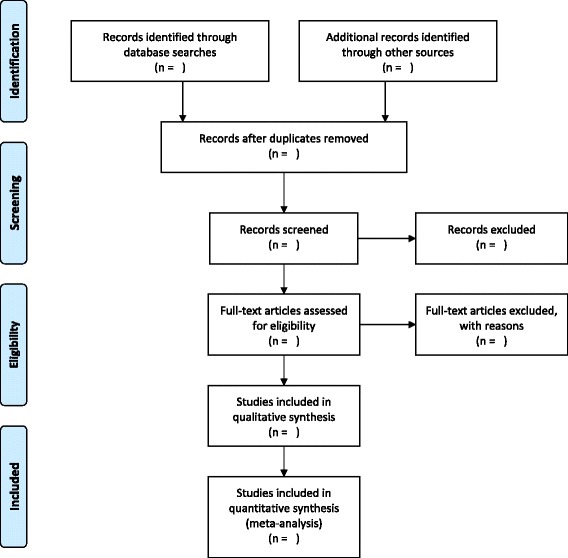
PRISMA flow diagram

We will search EMBASE and MEDLINE via Ovid SP, PubMed, and the Cochrane Library via Wiley Online Library with no restrictions on date/time, language, document type, and publication status. Keywords will be collected through experts’ opinion, literature review, controlled vocabulary (Medical Subject Headings = MeSH and Excerpta Medica Tree = EMTREE) and by reviewing the primary search results. Also, to retrieve cohort studies, we will use the search filter developed by the BMJ Evidence Centre. Search strategies developed with the assistance of a medical information specialist were reported in the Appendix. The process of study selection is shown in a PRISMA flow diagram (Fig. 1).

### Inclusion and exclusion criteria

We will identify prospective and retrospective cohort studies. Physical performance will be defined as upper extremity strength (e.g., grip strength), lower extremity strength (e.g., isometric or isotonic knee extension), walking speed, or other performance scale (e.g., short physical performance battery). We will include male and female participants of any age in community settings who have a history of the following: myocardial infarction, or coronary revascularization (coronary artery bypass grafting, percutaneous transluminal coronary angioplasty, or coronary artery stent), angina pectoris, heart failure, heart transplant, or coronary artery disease defined by angiography. We will include studies that enrolled participants with these histories confirmed by the medical record or administrative data.

We will exclude from the analysis those participants who have dementia, low vision or blindness, orthopedic surgery (e.g., hip or knee replacement and spinal surgery), and paralysis due to stroke. If such histories are not available in the paper, we will contact the authors of the study to obtain the missing information.

### Type of main outcomes

1. Mortality due to all cause
2. Mortality due to cardiovascular event
3. Re-hospitalization due to cardiovascular event

### Data extraction and management

We will use a data extraction form for the study information and outcome data. Six review authors (SY, TY, SN, TI, YS, and MO) will be divided into three teams (SY and TY, SN and TI, and YS and MO). They will independently extract study information from the included studies, as follows:

1. Methods: study design, study setting, duration of follow-up, and statistical analysis
2. Subjects: sample size, mean age, sex, history of stroke, history of orthopedics, and history of cancer
3. Outcome measures: physical performance test, concomitant medications, and excluded medications
4. Outcomes: main outcomes specified and collected and time points reported
5. Notes: funding for trial and notable conflicts of interest of trial authors

If the authors disagree with each other in the teams, we will resolve disagreements by discussion or by involving a third person (EO). One review author (SY) will transfer data from a data extraction form to the Review Manager file. We will double-check whether the data is entered correctly by comparing the presented data in the systematic review and research reports data. A second review author (RM) will randomly check the extracted data and characteristics against the source in order to ensure accuracy.

### Assessment of risk of bias

Two review authors (SY and TY) will independently evaluate the risk of bias for each study according to the Risk of Bias Assessment Tool: for Non-Randomized Studies (RoBANS) [16]. We will resolve any disagreements by discussion or by involving other authors (EO and RM). We will evaluate the risk of bias assessment tool, as follows:

1. Selection of participants
2. Confounding variables
3. Measurement of exposure
4. Blinding of outcome assessments
5. Incomplete outcome data
6. Selective outcome reporting

We will assess each domain as high, low, or unclear risk of bias and determine our judgements using criteria from the *Cochrane Handbook* [17].

## Data synthesis and analysis

We will perform the meta-analysis independently by each physical performance scale (e.g., upper extremity strength or lower extremity strength). Participants will be divided into subgroups according to age (middle-aged < 65 years and elderly ≥ 65 years), diagnosis (CAD and heart failure), and follow-up time (up to 12 months and over 12 months). Data synthesis and analyses will be performed using Review Manager version 5.3. We will perform a meta-analysis using the hazard ratios after logarithmic transformation by using inverse-variance method. Furthermore, we will synthesize the hazard ratios adjusted by variables of age and sex at least because the physical performance scales are closely affected by that factors. If we are able to pool more than 10 trials, we will create and examine funnel plot asymmetry visually in order to explore publication bias. If there is a suspicion of publication bias, we will carry out a simulation to investigate the possible small-study effect. Heterogeneity will be assessed among included studies in each analysis using the chi-square test of heterogeneity and *I*^2^ statistic. Data from each study will be pooled using random effects modeling where appropriate. To examine the robustness of results, we will perform meta-analyses using fixed-effect models after attributing less weight to small trials. We will use these meta-analyses only if their results differ from those of random effects models. When an *I*^2^ score of >75 % is obtained, we will consider heterogeneity to be substantial.

## Discussion

To our knowledge, this will be the first meta-analysis to assess the association between physical performance and cardiovascular events in CAD patients. The main goals for CAD patients are to prevent re-admission due to cardiac events and improve mortality. Many cohort studies have demonstrated that physical performance testing is strongly associated with mortality and cardiac events for CAD patients. Furthermore, physical performance testing is a quick, safe, inexpensive, and reliable assessment tool. We hope that our findings may help to estimate the prognosis for CAD patients in clinical practice.

## Appendix

### Search strategies

A. EMBASE

1. Exp Heart Failure/OR Exp Ischemic Heart Disease/OR Exp Cardiomegaly/OR Exp Cardiomyopathy/ OR Exp Coronary Artery Disease/OR Coronary Artery Bypass Graft/OR Exp Coronary Blood vessel/OR Exp Heart Surgery/OR Exp Percutaneous Coronary Intervention/OR (Myocardial Ischemi$ OR Myocardial Infarct$ OR Cardiovascular Stroke OR Ischemic Heart Disease? OR Angina? OR ((Heart OR Cardiac OR Myocardial) adj2 (Failure? OR Surg$ OR Operat$ OR Resect$ OR Transplant$ OR Graft$ OR Massage?)) OR CHF OR CABG OR Coronary OR Aortocoronary OR Sinus Node Arter$ OR Intracardiac Surg$ OR Cardiosurg$ OR Annuloplast$ OR Valve Annul$ OR Cardiomyoplast$ OR Cardioplegia? OR Heart Bypass$ OR Ventric$ Bypass$ OR Cavopulmonary Anastomos$ OR Cavopulmonary Shunt? OR Heart Valv$ OR Cardiac Valv$ OR Aort$ Valv$ OR Mitral Valv$ OR Pulmonary Valv$ OR Tricuspid Valv$ OR ((Heart OR Myocardial OR Transmyocardial OR Trans Myocardial) adj2 Revasculari$) OR Internal Mammary Artery Implant$ OR Pericardiostom$ OR Pericardiectom$ OR Pericardectom$ OR Pericardiotom$ OR Pericardotom$ OR Pericardiocentes$ OR Pericardial Aspirat$ OR Pericardium Puncture? OR Rotational Atherectom$ OR Epicardi$ Mapping OR Heart Aneurysmectom$ OR Valvuloplast$ OR Valv$ Repair$ OR Valvotom$ OR Valvulotom$ OR ((Prosthe$ OR Artificial OR Mechanical) adj2 Valve?) OR Heart Enlargement? OR Enlarged Heart OR Cardiac Hypertroph$ OR Heart Hypertroph$ OR Cardiomyopath$ OR Ventric$ Hypertroph$ OR Atrial Dilatation OR Atrial Enlargement OR Atrial Hypertroph$ OR Atriomegaly OR Atrium Dilatation OR Atrium Enlargement OR Atrium Hypertroph$ OR Cardiac Muscle Hypertroph$ OR Heart Hyperplasia OR Heart Muscle Hypertroph$ OR Myocardial Hypertroph$ OR Myocardium Hypertroph$ OR Ventric$ Wall Thickness OR Myocardial Disease? OR Myocardiopath$ OR Adhalinopath$ OR Sarcoglycanopath$ OR Antopol Disease OR Ventric$ Dysplasia OR Trypanosomiasis OR Carditis OR Endocardial Fibroelastos$ OR Endomyocardial Fibroelastosis OR Endomyocardial Fibros$ OR Myocardial Ischemic Reperfusion Injur$ OR Myocarditi$ OR Obstructive Asymmetric Septal Hypertroph$ OR Heart Amyloidosis OR Heart Myopath$ OR Cardiac Amyloidosis OR Oculocraniosomatic).ti,ab.
2. Exp Leg/OR Exp Arm/AND (Strength OR Force OR Power).ti,ab.
3. Exp Walking/ OR Muscle Strength/OR (Postural Balance OR Musculoskeletal Equilibrium OR Postural Equilibrium OR Gait OR Gaits OR Stair? Navigation? OR ((Hand OR Hands OR Pinch OR Leg OR Legs OR Lower Extremit$ OR Lower Limb? OR Membrum Inferius OR Upper Extremit$ OR Upper Limb? OR Membrum Superius OR Muscle? OR Muscle? Dynamic OR Muscular OR Muscular Dynamic OR Arm OR Arms) adj (Strength OR Force OR Power)) OR Grip OR Grips OR Grasp? OR Walk$ OR Locomotion OR Ambulation OR (Physical adj (Balance OR Function OR Performance OR Strength OR Activit$))).ti,ab.
4. 2 OR 3
5. Exp cohort analysis/OR Exp longitudinal study/OR Exp prospective study/OR Exp follow up/OR Cohort$.tw.
6. 1 AND 4 AND 5
7. limit 6 to human
8. limit 7 to exclude medline journals

B. MEDLINE

1. Exp Heart Failure/OR Exp Myocardial Ischemia/OR Exp Cardiomegaly/OR Exp Cardiomyopathies/OR Exp Coronary Disease/OR Exp Coronary Artery Bypass/OR Exp Coronary Vessels/OR Exp Cardiac Surgical Procedures/OR Exp Percutaneous Coronary Intervention/OR (Myocardial Ischemi$ OR Myocardial Infarct$ OR Cardiovascular Stroke? OR Ischemic Heart Disease? OR Angina? OR ((Heart OR Cardiac OR Myocardial) adj2 (Failure? OR Surg$ OR Operat$ OR Resect$ OR Transplant$ OR Graft$ OR Massage?)) OR CHF OR CABG OR Coronary OR Aortocoronary OR Sinus Node Arter$ OR Intracardiac Surg$ OR Cardiosurg$ OR Annuloplast$ OR Valve Annul$ OR Cardiomyoplast$ OR Cardioplegia? OR Heart Bypass$ OR Ventric$ Bypass$ OR Cavopulmonary Anastomos$ OR Cavopulmonary Shunt? OR Heart Valv$ OR Cardiac Valv$ OR Aort$ Valv$ OR Mitral Valv$ OR Pulmonary Valv$ OR Tricuspid Valv$ OR ((Heart OR Myocardial OR Transmyocardial OR Trans Myocardial) adj2 Revasculari$) OR Internal Mammary Artery Implant$ OR Pericardiostom$ OR Pericardiectom$ OR Pericardectom$ OR Pericardiotom$ OR Pericardotom$ OR Pericardiocentes$ OR Pericardial Aspirat$ OR Pericardium Puncture? OR Rotational Atherectom$ OR Epicardi$ Mapping OR Heart Aneurysmectom$ OR Valvuloplast$ OR Valv$ Repair$ OR Valvotom$ OR Valvulotom$ OR ((Prosthe$ OR Artificial OR Mechanical) adj2 Valve?) OR Heart Enlargement? OR Enlarged Heart OR Cardiac Hypertroph$ OR Heart Hypertroph$ OR Cardiomyopath$ OR Ventric$ Hypertroph$ OR Atrial Dilatation OR Atrial Enlargement OR Atrial Hypertroph$ OR Atriomegaly OR Atrium Dilatation OR Atrium Enlargement OR Atrium Hypertroph$ OR Cardiac Muscle Hypertroph$ OR Heart Hyperplasia OR Heart Muscle Hypertroph$ OR Myocardial Hypertroph$ OR Myocardium Hypertroph$ OR Ventric$ Wall Thickness OR Myocardial Disease? OR Myocardiopath$ OR Adhalinopath$ OR Sarcoglycanopath$ OR Antopol Disease OR Ventric$ Dysplasia OR Trypanosomiasis OR Carditis OR Endocardial Fibroelastos$ OR Endomyocardial Fibroelastosis OR Endomyocardial Fibros$ OR Myocardial Ischemic Reperfusion Injur$ OR Myocarditi$ OR Obstructive Asymmetric Septal Hypertroph$ OR Heart Amyloidosis OR Heart Myopath$ OR Cardiac Amyloidosis OR Oculocraniosomatic).ti,ab.
2. Exp Lower Extremity/OR Exp Upper Extremity/ AND (Strength OR Force OR Power).ti,ab.
3. Postural Balance/OR Gait/OR Exp Muscle Strength/OR Muscle Strength Dynamometer/OR Exp Walking/OR (Postural Balance OR Musculoskeletal Equilibrium OR Postural Equilibrium OR Gait OR Gaits OR Stair? Navigation? OR ((Hand OR Hands OR Pinch OR Leg OR Legs OR Lower Extremit$ OR Lower Limb? OR Membrum Inferius OR Upper Extremit$ OR Upper Limb? OR Membrum Superius OR Muscle? OR Muscle? Dynamic OR Muscular OR Muscular Dynamic OR Arm OR Arms) adj (Strength OR Force OR Power)) OR Grip OR Grips OR Grasp? OR Walk$ OR Locomotion OR Ambulation OR (Physical adj (Balance OR Function OR Performance OR Strength OR Activit$))).ti,ab.
4. 2 OR 3
5. Exp cohort studies/ or Cohort$.tw. or Controlled clinical trial.pt.
6. Epidemiologic methods/
7. Limit 2 to yr = 1971-1988
8. 5 OR 7
9. 1 and 4 and 8
10. limit 9 to humans

C. PubMed

("Myocardial Ischemia"[tiab] OR "Myocardial Infarction"[tiab] OR "Cardiovascular Stroke"[tiab] OR "Cardiovascular Disease"[tiab] OR "Cardiovascular Events"[tiab] OR "Ischemic Heart Disease"[tiab] OR Angina[tiab] OR ((Heart[tiab] OR Cardiac[tiab] OR Myocardial[tiab]) AND (Failure[tiab] OR Surgery[tiab] OR Operation[tiab] OR Transplantation[tiab] OR Graft[tiab])) OR CHF[tiab] OR CABG[tiab] OR Coronary[tiab] OR Aortocoronary[tiab] OR "Intracardiac Surgery"[tiab] OR Cardiosurgery[tiab] OR Annuloplasty[tiab] OR "Valve Annuloplasty"[tiab] OR Cardiomyoplasty[tiab] OR Cardioplegia[tiab] OR "Heart Bypass"[tiab] OR "Ventricular Bypass"[tiab] OR "Cavopulmonary Shunting"[tiab] OR Pericardiostomy[tiab] OR Pericardiectomy[tiab] OR Pericardectomy[tiab] OR Pericardiotomy[tiab] OR Pericardotomy[tiab] OR "Pericardial Aspiration"[tiab] OR "Pericardium Puncture"[tiab] OR "Rotational Atherectomy"[tiab] OR Valvuloplasty[tiab] OR "Valve Repair"[tiab] OR Valvotomy[tiab] OR Valvulotomy[tiab] OR "Prosthetic Valve"[tiab] OR "Artificial Valve"[tiab] OR "Mechanical Valve"[tiab] OR "Heart Enlargement"[tiab] OR "Enlarged Heart"[tiab] OR "Cardiac Hypertrophy"[tiab] OR "Heart Hypertrophy"[tiab] OR Cardiomyopathy[tiab] OR "Cardiac Muscle Hypertrophy"[tiab] OR "Heart Muscle Hypertrophy"[tiab] OR "Myocardial Hypertrophy"[tiab] OR "Myocardium Hypertrophy"[tiab] OR "Ventricular Wall Thickness"[tiab] OR "Myocardial Disease"[tiab] OR Antopol Disease[tiab] OR Trypanosomiasis[tiab] OR Carditis[tiab] OR "Endocardial Fibroelastosis"[tiab] OR "Endomyocardial Fibroelastosis"[tiab] OR "Endomyocardial Fibrosis"[tiab] OR "Myocardial Ischemic Reperfusion Injury"[tiab] OR Myocarditis[tiab] OR "Heart Myopathy"[tiab]) AND ("Postural Balance"[tiab] OR "Postural Equilibrium"[tiab] OR Gait[tiab] OR Gaits[tiab] OR "Stair Navigation"[tiab] OR ((Hand[tiab] OR Hands[tiab] OR Pinch[tiab] OR Leg[tiab] OR Legs[tiab] OR (Lower[tiab] AND Extremit*[tiab]) OR (Lower[tiab] AND Limb*[tiab]) OR (Upper[tiab] AND Extremit*[tiab]) OR (Upper[tiab] AND Limb*[tiab]) OR Muscle*[tiab] OR (Muscle*[tiab] AND Dynamic[tiab]) OR Muscular[tiab] OR Arm[tiab] OR Arms[tiab]) AND (Strength[tiab] OR Force[tiab] OR Power[tiab])) OR Grip[tiab] OR Grips[tiab] OR Grasp*[tiab] OR Walk*[tiab] OR Locomotion[tiab] OR Ambulation[tiab] OR (Physical[tiab] AND (Balance[tiab] OR Function[tiab] OR Performance[tiab] OR Strength[tiab] OR Activit*[tiab]))) AND ("Cohort Studies"[MeSH] OR Cohort*[All Fields] OR "Controlled Clinical Trial"[pt] OR Prospective[tiab] OR Follow-Up[tiab] OR "Follow Up"[tiab] OR Followup[tiab]) NOT MEDLINE[sb]

*D. Cochrane Library*

#1 MeSH descriptor: [Heart Failure] explode all trees

#2 MeSH descriptor: [Myocardial Ischemia] explode all trees

#3 MeSH descriptor: [Cardiomegaly] explode all trees

#4 MeSH descriptor: [Cardiomyopathies] explode all trees

#5 MeSH descriptor: [Coronary Disease] explode all trees

#6 MeSH descriptor: [Coronary Artery Bypass] explode all trees

#7 MeSH descriptor: [Coronary Vessels] explode all trees

#8 MeSH descriptor: [Cardiac Surgical Procedures] explode all trees

#9 MeSH descriptor: [Percutaneous Coronary Intervention] explode all trees

#10 (Myocardial Ischemi* or Myocardial Infarct* or Cardiovascular Stroke* or Ischemic Heart Disease* or Angina* or ((Heart or Cardiac or Myocardial) near/2 (Failure* or Surg* or Operat* or Resect* or Transplant* or Graft* or Massage*)) or CHF or CABG or Coronary or Aortocoronary or Sinus Node Arter* or Intracardiac Surg* or Cardiosurg* or Annuloplast* or Valve Annul* or Cardiomyoplast* or Cardioplegia* or Heart Bypass* or Ventric* Bypass* or Cavopulmonary Anastomos* or Cavopulmonary Shunt* or Heart Valv* or Cardiac Valv* or Aort* Valv* or Mitral Valv* or Pulmonary Valv* or Tricuspid Valv* or ((Heart or Myocardial or Transmyocardial or Trans Myocardial) near/2 Revasculari*) or Internal Mammary Artery Implant* or Pericardiostom* or Pericardiectom* or Pericardectom* or Pericardiotom* or Pericardotom* or Pericardiocentes* or Pericardial Aspirat* or Pericardium Puncture* or Rotational Atherectom* or Epicardi* Mapping or Heart Aneurysmectom* or Valvuloplast* or Valv* Repair* or Valvotom* or Valvulotom* or ((Prosthe* or Artificial or Mechanical) near/2 Valve*) or Heart Enlargement* or Enlarged Heart or Cardiac Hypertroph* or Heart Hypertroph* or Cardiomyopath* or Ventric* Hypertroph* or Atrial Dilatation or Atrial Enlargement or Atrial Hypertroph* or Atriomegaly or Atrium Dilatation or Atrium Enlargement or Atrium Hypertroph* or Cardiac Muscle Hypertroph* or Heart Hyperplasia or Heart Muscle Hypertroph* or Myocardial Hypertroph* or Myocardium Hypertroph* or Ventric* Wall Thickness or Myocardial Disease* or Myocardiopath* or Adhalinopath* or Sarcoglycanopath* or Antopol Disease or Ventric* Dysplasia or Trypanosomiasis or Carditis or Endocardial Fibroelastos* or Endomyocardial Fibroelastosis or Endomyocardial Fibros* or Myocardial Ischemic Reperfusion Injur* or Myocarditi* or Obstructive Asymmetric Septal Hypertroph* or Heart Amyloidosis or Heart Myopath* or Cardiac Amyloidosis or Oculocraniosomatic):ti,ab

#11 #1 or #2 or #3 or #4 or #5 or #6 or #7 or #8 or #9 or #10

#12 MeSH descriptor: [Lower Extremity] explode all trees

#13 MeSH descriptor: [Upper Extremity] explode all trees

#14 (Strength or Force or Power):ti,ab

#15 MeSH descriptor: [Postural Balance] explode all trees

#16 MeSH descriptor: [Gait] explode all trees

#17 MeSH descriptor: [Muscle Strength] explode all trees

#18 MeSH descriptor: [Muscle Strength Dynamometer] explode all trees

#19 MeSH descriptor: [Walking] explode all trees

#20 (Postural Balance or Musculoskeletal Equilibrium or Postural Equilibrium or Gait or Gaits or Stair* Navigation* or ((Hand or Hands or Pinch or Leg or Legs or Lower Extremit* or Lower Limb* or Membrum Inferius or Upper Extremit* or Upper Limb* or Membrum Superius or Muscle* or Muscle* Dynamic or Muscular or Muscular Dynamic or Arm or Arms) near/1 (Strength or Force or Power)) or Grip or Grips or Grasp* or Walk* or Locomotion or Ambulation or (Physical near/1 (Balance or Function or Performance or Strength or Activit*))):ti,ab

#21 (#12 or #13) and #14

#22 #15 or #16 or #17 or #18 or #19 or #20 or #21

#23 #11 and #22

## Abbreviations

ACROBAT-NRSI: A Cochrane Risk Of Bias Assessment Tool: for Non-Randomized Studies of Interventions
CAD: coronary artery disease
MOOSE: Meta-analysis of Observational Studies in Epidemiology
